# Assessment of a model based optimization engine for volumetric modulated arc therapy for patients with advanced hepatocellular cancer

**DOI:** 10.1186/s13014-014-0236-0

**Published:** 2014-10-28

**Authors:** Antonella Fogliata, Po-Ming Wang, Francesca Belosi, Alessandro Clivio, Giorgia Nicolini, Eugenio Vanetti, Luca Cozzi

**Affiliations:** Oncology Institute of Southern Switzerland, Via ospedale, 6504 Bellinzona, Switzerland; Department of Radiation Oncology, Cheng-Ching General Hospital, Taichung, Taiwan

**Keywords:** Knowledge based planning, RapidPlan, RapidArc, Liver cancer

## Abstract

**Background:**

To evaluate in-silico the performance of a model-based optimization process for volumetric modulated arc therapy (RapidArc) applied to hepatocellular cancer treatments.

**Patients and methods:**

45 clinically accepted RA plans were selected to train a knowledge-based engine for the prediction of individualized dose-volume constraints. The model was validated on the same plans used for training (closed-loop) and on a set of other 25 plans not used for the training (open-loop). Dose prescription, target size, localization in the liver and arc configuration were highly variable in both sets to appraise the power of generalization of the engine. Quantitative dose volume histogram analysis was performed as well as a pass-fail analysis against a set of 8 clinical dose-volume objectives to appraise the quality of the new plans.

**Results:**

Qualitative and quantitative equivalence was observed between the clinical and the test plans. The use of model-based optimization lead to a net improvement in the pass-rate of the clinical objectives compared to the plans originally optimized with standard methods (this pass-rate is the frequency of cases where the objectives are respected vs. the cases where constraints are not fulfilled). The increase in the pass-rate resulted of 2.0%, 0.9% and 0.5% in a closed-loop and two different open-loop validation experiments.

**Conclusions:**

A knowledge-based engine for the optimization of RapidArc plans was tested and lead to clinically acceptable plans in the case of hepatocellular cancer radiotherapy. More studies are needed before a broad clinical use.

## Background

The radiation-oncology treatment planning process, is a step where many information shall merge leading to the most appropriate technique and dose distribution for all individual patients. Different levels of “knowledge” contribute to decision making. The determination of the appropriate dose-volume constraints is a problem that could be “modeled” if the “features” causing inter-patient variability could be converted into mathematical methods. These features include the geometric, anatomical and dosimetric characteristics of the treatment plans. The group of the Duke University developed so-called knowledge-based methods to solve this and applied them to clinical experiments and to inter-institutional validation [1-5]. The Washington University group developed similar architectures to appraise the quality of treatment plans and to predict appropriate dose-volume constraints [6,7]. Others focused on the possibility to automate the optimization process and to test this in-silico or in clinical trials with the final aim to develop data-driven, machine-based systems supporting the human clinical decision [8-14].

Aim of the present study is the evaluation of a knowledge-based dose-volume constraints prediction engine recently implemented in a commercial treatment planning system (TPS) partially based on the investigations of the Duke’s group and further developed. The questions to be addressed were: i) can such a predictive model be built and lead to acceptable results for a real clinical problem? ii) can a model be built with a reasonably limited dataset of training patients? iii) can the model be reliable when no special selection criteria are applied to generate the training, i.e. including cases with the only requirement to be clinically acceptable?.

The case of advanced stage hepatocellular carcinoma (HCC) was chosen (high variability in tumor size and position in the liver). All the investigation was performed in the arena of volumetric modulated arc therapy (VMAT).

## Material and methods

### The knowledge-based environment

A new optimization engine was introduced in the Eclipse TPS (Varian Medical Systems, Palo Alto, USA) in the release 13.5. This is made of three main components: a model building and training engine; a model-based dose-volume histogram (DVH) and automated constraints prediction tool; a new VMAT and IMRT optimization algorithm to manage the above.

The sequence of main steps necessary to generate a model is as follows:

- Selection of a set of training plans. No specific requirements are mandatory to be candidate for training. The strategy adopted was to create a “universal” model for HCC and the only requirement was to select plans accepted for clinical treatment.
- Association of these plans to a model layout where target and organs at risk ontology, dose prescriptions and some descriptive elements can be defined.
- Definition of the type of constraints to be generated per each structure (points vs. lines, priorities, user defined vs. fully automated).
- Dosimetric and geometrical data extraction from the patient database to the model engine.
- Model training. Based on principal component (PC) analysis methods [2,3,15,16].
- Model publication and validation.

**Figure 1:**
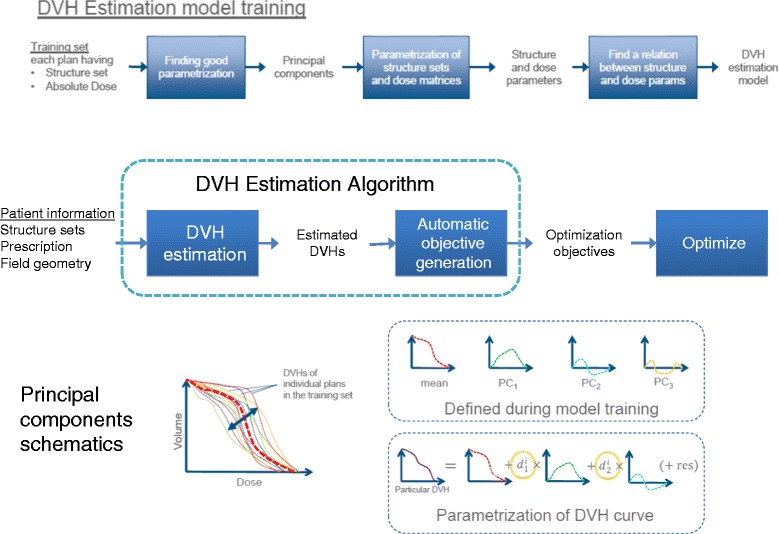
**A schematic representation of the model determination steps and of the PC method applied to DVH.**

Figure 1 shows a schematic representation of the model determination steps and of the PC method applied to DVH. The assumption is that any DVH can be represented as a combination of the average DVH over a population plus a sum of some weighted PC and a residual. The first PC is determined by maximizing the variance of the training set it can explain; any consecutive PC is chosen so that the residual variance is further accounted for. The features used to build the model primarily include geometric characteristic of the various structures, as well as their mutual position and their relationships with the treatment fields. These are modeled by constructing a Geometry-Based Expected Dose (GED) which evaluates the distance between each structure and the target surface by means of the amount of dose that each target contributes to an organ for the current field geometry. The final prediction model is built as a combination of the PC and regression techniques for the in-field region of any OAR and a mean and standard deviation model on the DVH fo the other OAR regions. The PC is applied to the GED and DVH to find the main component scores.

**Figure 2:**
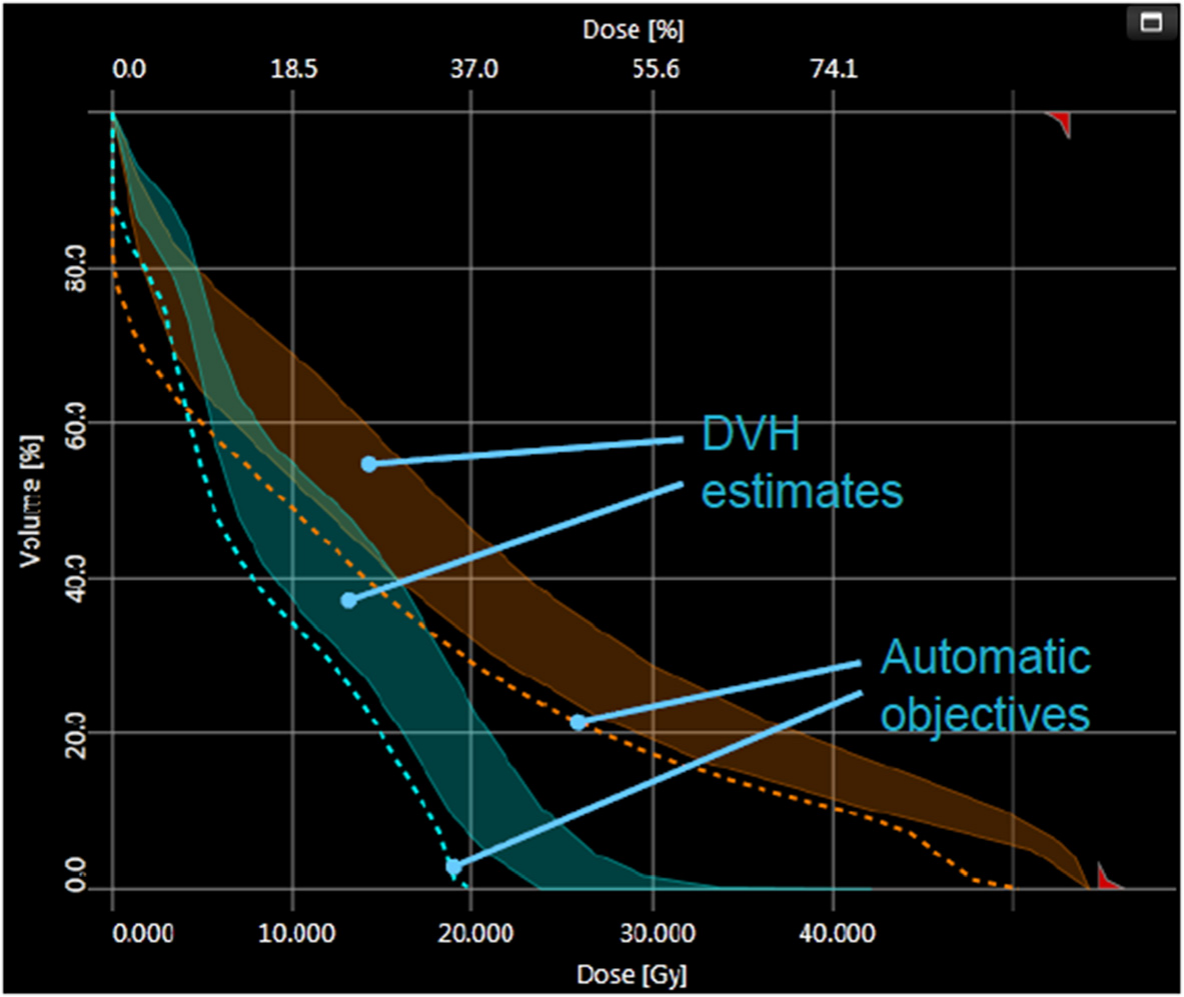
**Examples of the model-based predictive objectives with the estimate range and automatic objectives (line objectives in this example).**

A trained model, once made “public”, can be used to perform predictive estimation of the DVHs for any given new test case and, from these, to determine the planning constraints. The DVH prediction workflow is described as:

- Selection of a knowledge-based model
- Matching of structure names if the ontology mapping is not complete
- Prediction of a range of possible DVH for each of the structures present in both the plan and the model.
- Automatic generation of the dose-volume constraints based on the rules from the model configuration. With a fully automatic procedure, these are located below the lower limit of a prediction range generated from the most probable DVH curve by adding and subtracting a variation curve. This corresponds to 1 standard deviation for the out-of-field region. For the in-field region this is constructed by adding in quadrature the DVH PC multiplied by the standard error related to the model regression. Also the priorities are defined by the prediction engine and account for a basic balance between all possible trade-offs. All point constraints and priorities can be modified during optimization.
  Figure 2 exemplifies the resulting model-based predictive objectives with the estimate range and automatic objectives (line objectives in this example).

### Patient selection

A group of 145 HCC patients was identified for the study. All patients were managed in compliance with the Helsinki Declaration. Ethical approval for retrospective and in-silico studies was obtained by the institute’s ethical committee and patients provided their consent for data manipulation. RapidArc plans were approved and used for clinical treatment of each patient. Details of plans and treatment outcome have been described in detail in [17-19]. From this library, 45 patients were selected for the model training and other 25 were used for the validation experiments. The selection of the two groups was done to proportionally represent the dose prescription levels and the target volumes observed in the main population. Concerning the dose, the patients were chosen as follows: 55% with 60 Gy or more, 50% with 40-45 Gy and 5% with less than 40 Gy; in all cases the dose was prescribed at 2Gy/fractions. Target volume was chosen uniformly though the range of the population. Average target volume was 596 ± 538 cm^3^ (range: 54-2188 cm^3^) for the model and 697 ± 493 cm^3^ (79-2217 cm^3^) for the validation set. No other specific criteria were applied. The number of partial arcs used for the clinical plans ranged from 2 to 4 and non-coplanar setting was applied for at least one arc in 40/45 cases in the model-set and in 23/25 of the test cases. All plans used for the training and for the validation phases were accepted and used for treatment.

### Validation experiments

The system produces a summary about the training process with some quality metrics. In the results, the model goodness of estimation will be reported. This is the mean squared error between original and estimate data from a built-in cross validation where the training set is divided in 10 parts; 10 models are trained and each model is tested against a different tenth of the data points that are left-out. The average of the validation statistics generated during the 10 rounds is reported and helps to evaluate how the model is able to estimate plans not used in the training. The regression model’s parameters average chi square measures the quality of the regression model. This value is linked to the Pearson’s chi square and is measured from the residual difference between the original data and the estimation. The closer to one the value the better is the quality of the regression model.

The whole estimation model’s fit describes how well the estimation model represents the training plans in the regression line. Results proximal to 1 suggest better quality.

The training report includes also information about the structures or patients that are found to be potential outliers based on some numerical metrics. In principle, structures can be identified as outliers if they significantly differ in contouring or in dose features from the rest of the population. Each candidate should be analyzed individually and, if judged truly different from the population, might be excluded from the training set.

Cases candidate to be outlier in a model can be basically of two different types: geometric or dosimetric. In geometric potential outliers, the structure contours significantly differ from the rest of the population and might indicate the use of different contouring protocols or even more basic issues. In dosimetric potential outliers, the dose trade-offs are significantly different from the rest of the population. This might indicate different planning strategies (or techniques), different acceptance criteria or, even, presence of sub-optimal plans.

Several metrics have been implemented to monitor and to quantify the presence of potential outlieres. Among these, the Cook’s distance (a standard statistical tool) suggests the presence of highly influencial points in the regression and suggests the need of careful evaluation of the data. Reporting threshold have been set to 4. The studentized residual describes the difference between actual and estimated parameters for individual training cases. The metrics is normalized by dividing the residuals by standard deviation. In addition studentized residual tries to take account that for more influential points, smaller residual is significant because the case has larger power to turn the residual towards itself.

Three sets of validation experiments were conducted:

i) Closed-loop: re-optimization of the 45 training cases using as optimization constraints the DVH estimated by the model. Aim of this phase is to understand if the prediction tool is capable to guarantee the same clinical quality of plans for the same cases used to build it. A positive validation is a pre-requisite.
ii) Open-loop I: optimization of the 25 test cases using the model-predicted DVH for the same beam geometry settings used for the clinical plans. A positive validation implies the capability of the prediction tool to generalize to un-known cases presented with a geometrical plan setting consistent with the training.
iii) Open-loop II: optimization of the 25 test cases using a simpler arrangement with 2 coplanar partial arcs. A positive validation implies the further capability of the system to generalize the DVH prediction when the geometry of the arcs is different from what used for the training.
  During the interactive phase of the optimization process, only the objective’s priorities were adjusted if judged needed to better balance between them. The starting point of the priorities was given by the model prediction. This did not affected the optimization time (no pauses were introduced) and was performed only if some of the structures were not responding or were too far from the constraints.
  In all cases the Acuros-XB photon dose calculation algorithm (v. 13.5) was applied with a dose matrix resolution of 2.5 mm. All plans were normalized to the mean dose to PTV as per institutional policy in clinical routine and in compliance to ICRU recommendations. Standard DVH quantitative analysis was performed to appraise the quality of the model-based optimized plans versus the clinically accepted plans. A total of 8 dose-volume endpoints were analyzed for each of the two groups of patients for target volume (PTV) and organs at risk (OAR). These were equal to what used for the clinical acceptance of the original plans and included: D_1cm3_ < 45 Gy to the spine, V_15Gy_ < 35% for both kidneys, V_36Gy_ < 5% for the stomach, D_1cm3_ < 60 Gy and V_55Gy_ < 30% for the esophagus, V_30Gy_ < 30% for the normal liver (liver-PTV) and D_98%_ > 90% for the PTV.

## Results

**Figure 3:**
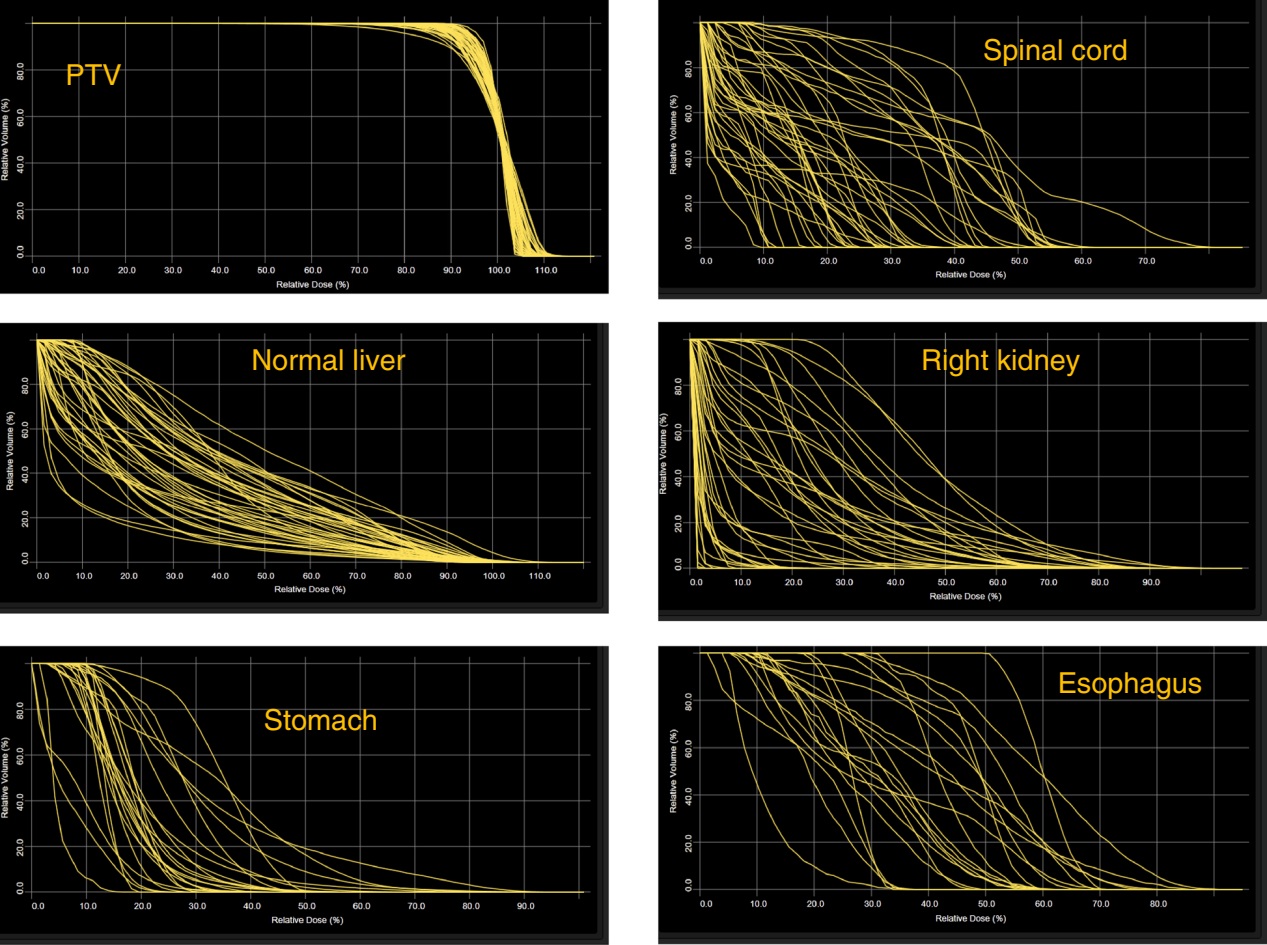
**The DVH for the population of 45 training patients for the PTV and OARs.**

**Figure 4:**
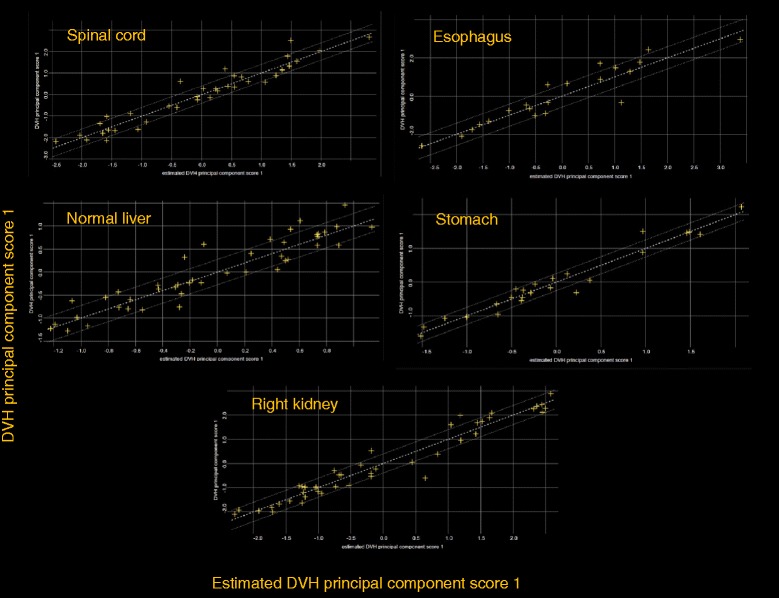
**Scatter plot and regression lines for some of the various principal-component analysis.**

Figure 3 shows the DVH for the population of 45 training patients for the PTV and different OARs. It is evident the large variance in the dataset. Figure 4 shows the scatter plots of the DVH first PC vs. the corresponding estimated one for some OARs; lines represents 1 SD. Narrow distributions correlate to better quality of the prediction power of the model. Points falling outside the lines might be outlier candidates. The time needed to extract the data to the model (dose distributions, geometric data for the beams and structure volume information) was about 10-15 seconds per patient. Model training took less than 2 minutes. The total mean model goodness of estimation (the mean squared error of the residuals) resulted 0.007 ± 0.005. Table 1 presents some of the training quality metrics from the system report. For some of the structures, the complexity of the problem would have required more cases although the estimation fit and the mean squared error between original and estimated data do not reflect this problem. The ideal number of cases is estimated by the training engine to be roughly equal to 5 times the number of parameters used for the regression model. An insufficient number of cases does not imply automatically a failure in the training but should be evaluated in relation to the quality of the validation. Table 2 reports some details about the number of candidate outliers per structure and the average of the pertinent metrics. The majority of cases identified as potential outliers presented a high Cook’s distance, i.e. have high influence in the determination of the regression coefficient. In a limited number of cases (12), some outliers presented also a large studentized residual value, this is a metric indicating that for more influential points, even small residuals are significant in the regression. Both categories do not suggest that the cases should be rejected but that might deserve some consideration if the validation of the model would fail. All cases were individually analyzed and none resulted characterized by evident errors or discrepancies with respect to the clinical protocol applied for treatment. Rather, the great variation in target shape and position (as well as dose prescription) can justify their presence. Data shown in Figure 4 confirm that there is no evidence of remarkably different cases but rather some “uniform” dispersion of the data over the range. This was one of the reasons to keep all potential outliers in the training set.

**Table 1:** **Summary the model goodness statistics**

	**Number of structures in the model**	**Recommended number of structures needed for training**	**Regression model’s parameters average chi square**	**Whole estimation model fit**	**MSE between original and estimate**
Small bowel	39	-	1.015	0.865	0.010
Left kidney	45	-	1.094	0.867	0.009
Right kidney	45	-	1.125	0.816	0.004
Left lung	35	60	1.169	0.860	0.002
Right lung	35	54	1.306	0.947	0.002
Esophagus	21	30	1.147	0.857	0.017
Normal liver	45	-	1.071	0.849	0.002
Spinal cord	45	-	1.567	0.891	0.008
Stomach	24	42	1.336	0.923	0.011

*MSE* mean squared error.

**Table 2:** **Analysis of outliers**

**Organ**	**Number of structures in the model**	**Number of potential outliers**	**Reason**	**Average score**
Small bowel	39	5	Cook’s distance: 5	11.5 ± 8.9
			SSR: 2	4.1 ± 0.9
Left kidney	45	9	Cook’s distance: 9	11.5 ± 6.1
			SSR: 4	4.0 ± 0.6
Right kidney	45	1	Cook’s distance: 1	4.6
Left lung	35	19	Cook’s distance: 19	19.1 ± 13.2
Right lung	35	13	Cook’s distance: 12	7.9 ± 5.7
			SSR: 3	4.0 ± 1.4
Esophagus	21	6	Cook’s distance: 2	5.3 ± 0.6
			SSR: 6	3.7 ± 0.8
Normal liver	45	7	Cook’s distance: 7	11.2 ± 9.3
Spinal cord	45	6	Cook’s distance: 6	8.2 ± 2.8
			SSR: 2	3.4 ± 0.0
Stomach	24	6	Cook’s distance: 3	6.5 ± 2.9
			SSR: 4	3.3 ± 0.5

*SSR* significant studentized residual.

**Figure 5:**
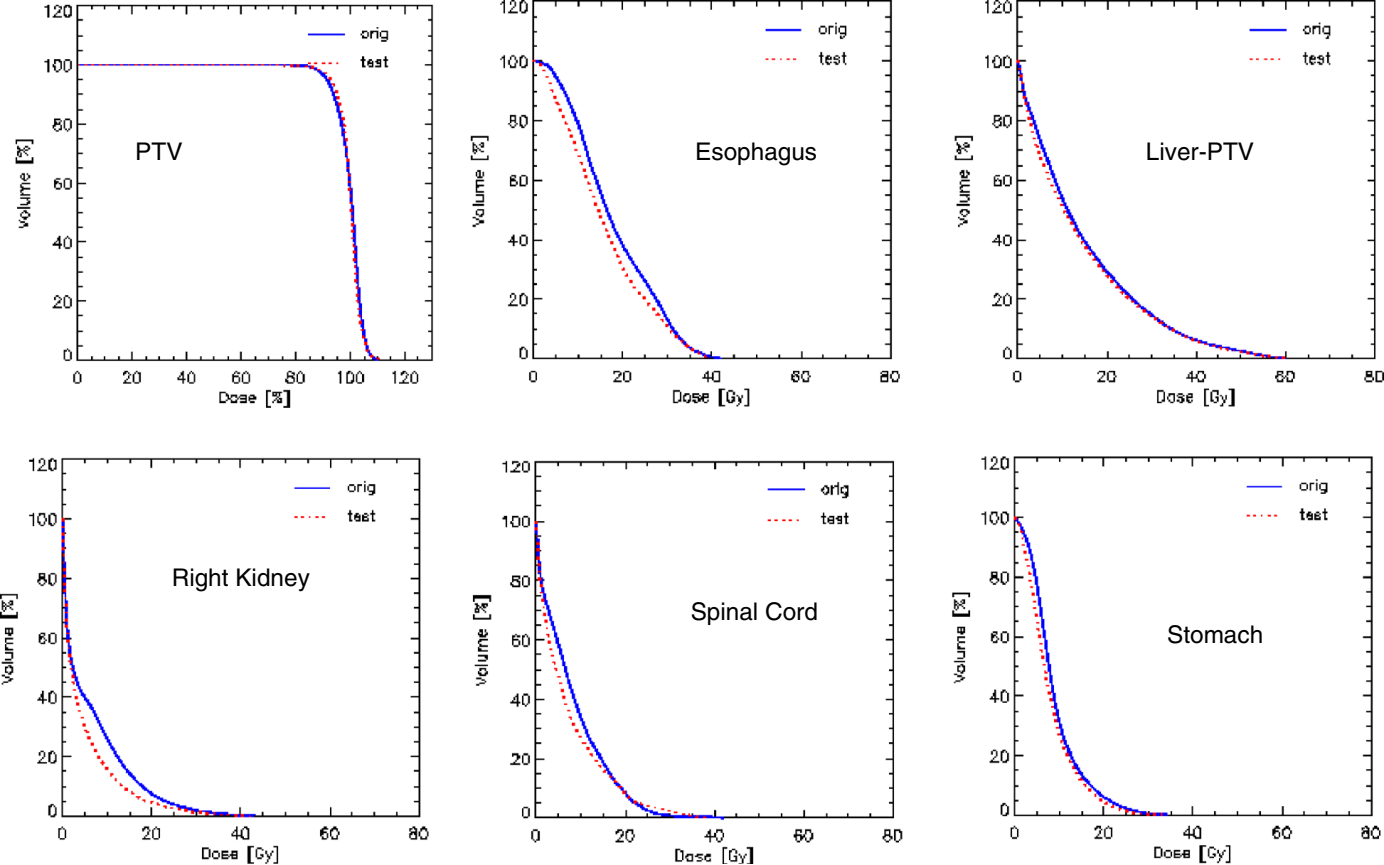
**Average DVH for the closed-loop validation experiment.** The Orig lines are for the clinical plans while the Test lines for the model-based optimization.

**Figure 6:**
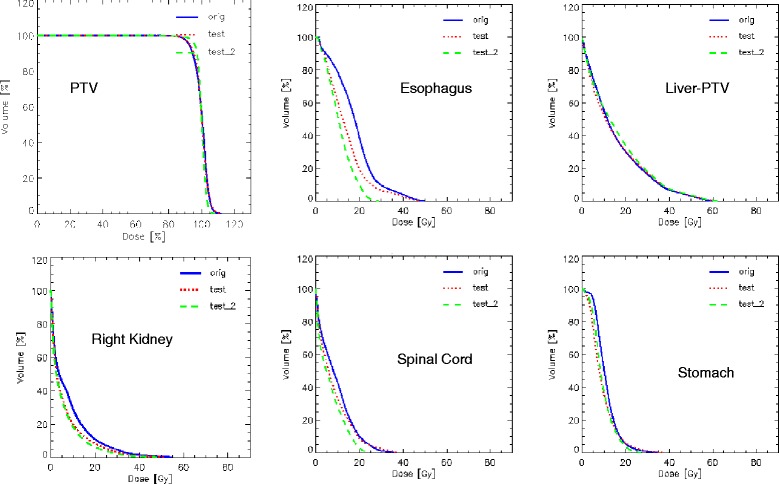
**Average DVH for the two open-loop validation experiments.** The Orig lines are for the clinical plans, the Test lines for the model-based optimization with the same non-coplanar geometry, and the Test_2 lines for the simplified, coplanar only arc setting.

**Figure 7:**
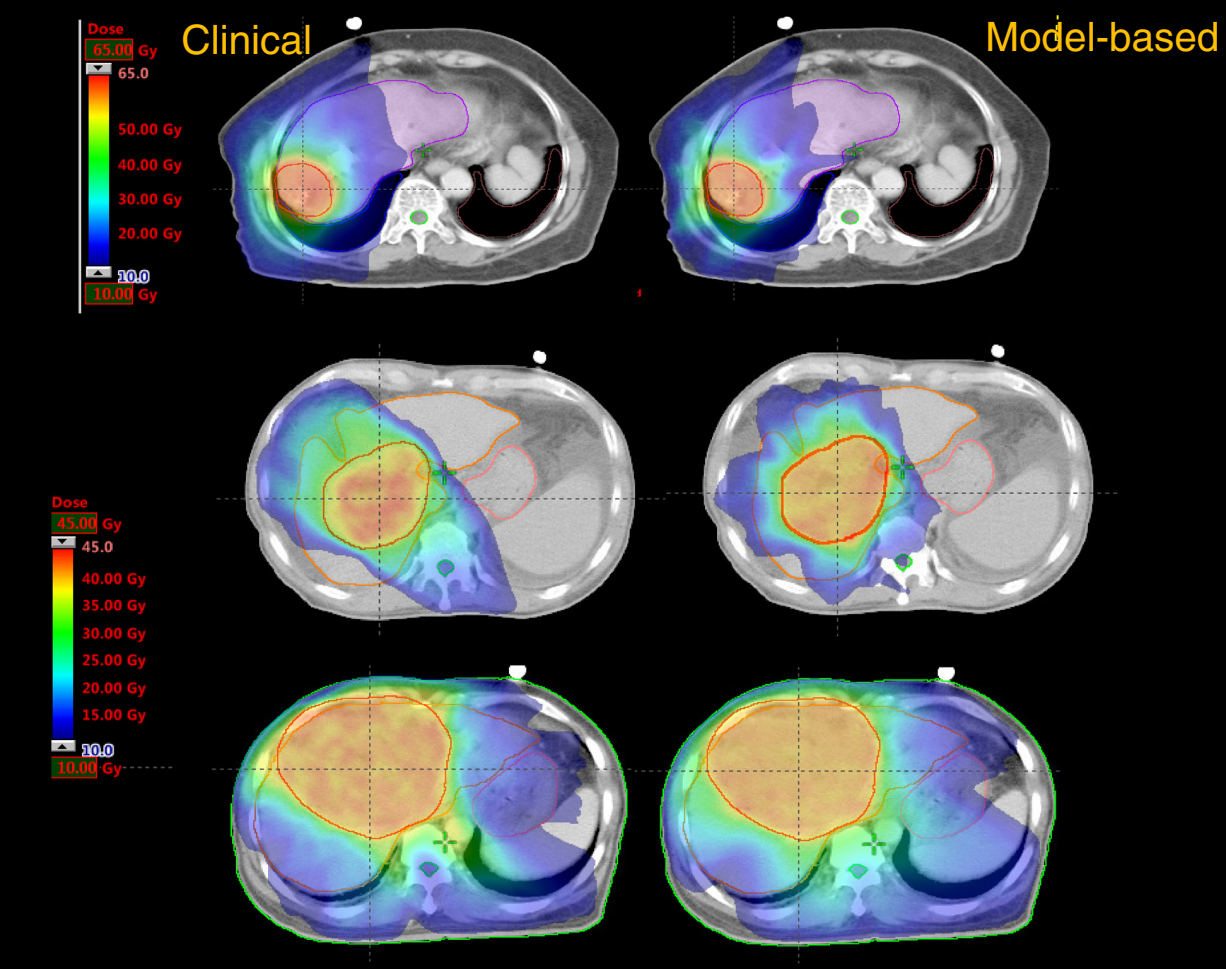
**Shows the axial isodose distributions for 3 examples from the open-loop validation.**

Figure 5 shows the average DVHs for the PTV and OARs for the clinical and for the re-optimized cases in the close-loop while Figure 6 shows the same for the 2 open-loop validations. DVH for the PTV are shown in % while in Gy for the OARs. Table 3 summarizes the DVH analysis. All planning objectives were on average met, in both clinical and test plans, with some limited individual violations. Model-based plans resulted on average improved with respect to the clinical cases, and the maximum value in the ranges is always higher for clinical than for test plans. Table 4 further expands the report on DVH analysis. Figure 7 shows the axial isodose distributions for 3 examples from the open-loop validation. The time needed to generate the estimated DVH for the test cases was about 15 seconds.

**Table 3:** **Summary the DVH analysis for the clinical cases of the closed-loop and the two open-loop validations**

**Model validation on the training set of 45 patients**	**Objective**	**Clinical plans**	**Closed-loop validation**		**p**
PTV: V_98%_ [%]	>90%	97.2 ± 0.9	96.8 ± 1.4	-	NS
		[94.9-98.9]	[91.4-98.7]		
Normal liver: V_30Gy_ [%]	<30%	14.7 ± 11.4	14.2 ± 9.6	-	NS
		[0.0-51.4]	[0.0-36.8]		
Spine: D_1cm3_ [Gy]	<45 Gy	15.6 ± 8.0	15.1 ± 9.0	-	<0.05
		[3.9-45.3]	[2.7-40.4]		
Left kidney V_15Gy_ [%]	<35%	1.3 ± 6.2	0.6 ± 2.6	-	<0.01
		[0.0-39.2]	[0.0-14.6]		
Right kidney V_15Gy_ [%]	<35%	14.3 ± 17.8	8.1 ± 10.8	-	<0.01
		[0.0-77.4]	[0.0-39.9]		
Stomach V_35Gy_ [%]	<5%	0.1 ± 0.7	0.1 ± 0.6	-	NS
		[0.0–3.3]	[0.0-2.8]		
Esophagus: D_1cm3_ [Gy]	<60 Gy	25.4 ± 8.5	23.4 ± 8.7	-	<0.10
		[12.5-39.9]	[10.4-37.4]		
Esophagus: V_30Gy_ [%]	<30%	0.0 ± 0.0	0.0 ± 0.0	-	NS
		[0.0-0.0]	[0.0-0.0]		
**Validation on the independent sample of 25 patients**	**Objective**	**Clinical plans**	**Open-loop Validation I**	**Open-loop Validation II**	
PTV: V_98%_ [%]	>90%	97.4 ± 0.9	96.8 ± 1.9	97.1 ± 2.6	I: <0.05
		[94.9-98.3]	[89.3-98.3]	[89.8-98.6]	II: NS
Normal Liver: V_30Gy_ [%]	<30%	16.2 ± 10.3	16.9 ± 9.9	18.0 ± 10.5	I: <0.05
		[0.0-36.3]	[0.0-35.1]	[0.0-39.5]	II: <0.01
Spine: D_1cm3_ [Gy]	<45 Gy	18.7 ± 7.4	17.6 ± 8.2	15.1 ± 5.5	I: <0.01
		[4.1-36.0]	[4.3-37.2]	[3.9-25.3]	II: <0.01
Left kidney V_15Gy_ [%]	<35%	1.4 ± 4.3	0.3 ± 1.4	0.01 ± 0.05	I: <0.01
		[0.0-19.6]	[0.0-6.9]	[0.0-0.3]	II: <0.01
Right kidney V_15Gy_ [%]	<35%	17.6 ± 18.5	12.8 ± 15.0	10.3 ± 10.8	I: <0.01
		[0.0-49.9]	[0.0-39.7]	[0.0-34.4]	II: <0.01
Stomach V_35Gy_ [%]	<5%	0.1 ± 0.3	0.3 ± 1.0	0.0 ± 0.0	I: <0.04
		[0.0-0.8]	[0.0-4.2]	[0.0-0.0]	II: NS
Esophagus: D_1cm3_ [Gy]	<60 Gy	25.7 ± 9.6	21.9 ± 9.8	17.3 ± 4.7	I: <0.01
		[11.9-46.9]	[0.5-46.1]	[10.8-25.1]	II: <0.01
Esophagus: V_30Gy_ [%]	<30%	0.0 ± 0.0	0.0 ± 0.0	0.0 ± 0.0	I: NS
		[0.0-0.0]	[0.0-0.0]	[0.0-0.0]	II: NS

*VxGy* Volume receiving at least XGy, *D*_*Y%*_ *(D*_*ycm3*_ *)* dose delivered to at least Y% (or cm^3^) of the volume. *NS* not significant. I: clinical vs. Open-loop I; II: clinical vs. Open-loop II.

**Table 4:** **Integration of the summary the DVH analysis for the clinical cases of the two open-loop validations**

**Validation on the independent sample of 25 patients**	**Clinical plans**	**Open-loop validation I**	**Open-loop validation II**	**p**
PTV: Mean [%]	100.0 ± 0.0	100.0 ± 0.0	100.0 ± 0.0	I: NS
	[0.0-0.0]	[0.0-0.0]	[0.0-0.0]	II: NS
PTV: D1% [%]	106.8 ± 2.4	107.6 ± 2.3	105.4 ± 1.0	I:<0.05
	[103.5-114.3]	[104.3-112.6]	[103.1-107.0]	II:<0.05
PTV: D99% [%]	90.0 ± 2.5	90.2 ± 3.7	92.8 ± 3.8	I:NS
	[82.6-93.5]	[79.4-94.8]	[80.4-95.9]	II:<0.05
PTV: HI	0.13 ± 0.04	0.12 ± 0.04	0.08 ± 0.02	I:NS
	[0.07-0.23]	[0.06-0.20]	[0.05-0.15]	II:<0.01
PTV: CI	1.11 ± 0.07	1.08 ± 0.05	1.05 ± 0.04	I:<0.05
	[1.05-1.21]	[1.01-1.13]	[1.00-1.11]	II:<0.01
Normal liver: Mean dose [Gy]	15.6 ± 5.5	15.4 ± 5.1	16.4 ± 5.4	I: NS
	[5.8-23.5]	[6.2-23.0]	[6.9-23.8]	II: <0.05
Spine: Mean dose [Gy]	9.2 ± 4.5	8.2 ± 4.9	6.6 ± 3.5	I: <0.05
	[1.0-19.7]	[0.9-19.9]	[0.8-15.5]	II: <0.01
Left kidney Mean dose [Gy]	3.9 ± 3.1	2.5 ± 2.1	2.0 ± 1.7	I: <0.01
	[0.2-12.7]	[0.2-9.2]	[0.1-7.5]	II: <0.01
Right kidney Mean dose [Gy]	7.9 ± 5.8	6.3 ± 4.8	5.6 ± 4.0	I: <0.01
	[0.7-18.9]	[0.6-14.6]	[0.8-12.6]	II: <0.01
Stomach Mean dose [Gy]	10.9 ± 2.9	9.2 ± 4.5	9.4 ± 3.5	I: <0.05
	[5.8-16.1]	[3.8-20.8]	[3.6-15.9]	II: <0.05
Esophagus: Mean dose [Gy]	17.8 ± 8.2	13.9 ± 7.4	10.9 ± 3.9	I: <0.05
	[4.9-38.2]	[3.8-34.1]	[4.6-18.0]	II: <0.01
Body-PTV: V10Gy [%]	17.1 ± 8.1	16.7 ± 7.9	16.8 ± 7.6	I: <0.10
	[2.6-31.5]	[2.6-32.9]	[3.1-32.3]	II: <0.10

For the PTV, conformity Index CI is defined as the ration between V95% and PTV. Homogeneity index HI is defined as (D5-D95)/Mean.

*VxGy* Volume receiving at least XGy, *D*_*Y%*_ *(D*_*ycm3*_ *)* dose delivered to at least Y% (or cm^3^) of the volume. *CI* Conformity Index, *HI* homogeneity Index, *NS* not significant. I: clinical vs. Open-loop I; II: clinical vs. Open-loop II.

Table 5 reports the values of the parameters that were violated either in the clinical or in the test plans.

**Table 5:** **Patterns of planning constraints violations in the closed loop validation**

**Patient**	**Parameter**	**Clinical plan**	**Closed-loop validation**	
1	Normal Liver V_30Gy_ < 30%	**31.7%**	**31.9%**	**-**
2	Normal Liver V_30Gy_ < 30%	**30.6%**	29.4%	-
3	Normal Liver V_30Gy_ < 30%	**34.8%**	29.8%	-
4	Spinal cord D_1cm3_ < 45Gy	**45.3%**	40.3%	-
	Left Kidney V_15Gy_ < 35%	**39.2%**	14.6%	-
	Right kidney V_15Gy_ < 35%	**53.8%**	**39.9%**	-
	Normal Liver V_30Gy_ < 30%	**51.4%**	**36.9%**	-
5	Right kidney V_15Gy_ < 35%	**77.4%**	**38.3%**	**-**
	Normal Liver V_30Gy_ < 30%	**31.8%**	23.3%	**-**
6	Right kidney V_15Gy_ < 35%	**41.6%**	19.4%	-
7	Right kidney V_15Gy_ < 35%	**43.6%**	26.7%	-
**Patient**	**Parameter**	**Clinical plan**	**Open-loop validation I**	**Open-loop validation II**
1	Normal Liver V_30Gy_ < 30%	**30.5%**	27.4%	**30.6%**
2	Right kidney V_15Gy_ < 35%	**46.9%**	**39.7%**	23.6%
3	Right kidney V_15Gy_ < 35%	**38.5%**	34.8%	34.4%
4	Normal Liver V_30Gy_ < 30%	29.3%	**31.0%**	29.9%
5	Right kidney V_15Gy_ < 35%	31.8%	**36.9%**	20.5%
6	Right kidney V_15Gy_ < 35%	**38.6%**	**36.6%**	21.5%
	PTV D_98%_ > 90%	94.8%	**89.3%**	**89.8%**
7	Normal Liver V_30Gy_ < 30%	**32.1%**	**32.5%**	**33.1%**
8	Normal Liver V_30Gy_ < 30%	**36.3%**	30.0%	**39.5%**
9	Right kidney V_15Gy_ < 35%	**49.9%**	26.9%	17.0%
10	Right kidney V_15Gy_ < 35%	**38.9%**	26.5%	25.0%
11	Right kidney V_15Gy_ < 35%	**46.3%**	25.6%	22.7%

In bold the parameters that were violated. The patients in the two groups are different.

### Closed-loop validation

Of the 360 dose-volume points analyzed (8 parameters for 45 patients), 11 points (3.1%) in 7 patients violated the constraints in the original clinical dataset; one patient had 4 simultaneous violations. Only 4 points (1.1%) in 3 patients failed to respect the objectives in the test set (and only 2 simultaneous violations in 1 patient). All 4 failures in the validation phase were also failures in the clinical plans.

### Open-loop validation I

9 of 224 dose-volume points (4.0%, 9 patients, 1 failure per patient, 6 in the right kidney and 3 in the healthy liver) failed to respect the constraints in the clinical dataset of 25 patients. This number was reduced to 6 failures (2.7%) in the model-based plans. Of these 3 (2 in the right kidney, 2 in the healthy liver) were already present in the clinical plans while 3 new failures (2 on the same patient) were observed in the right kidney and D_98%_ to the PTV (89.3% instead of 94.8% of the test clinical plan and 90% of the objective).

### Open-loop validation II

Only 4 of 224 dose-volume points (1.8%) failed in the test group (against the same 9 described above) in 4 patients. Three in the healthy liver and one in the D_98%_ to the PTV (89.8% instead of the 94.8% of the clinical and 90% of the objective).

Despite this was not a primary objective of the study, the net improvement in passing the clinical objectives for the model-based plans resulted modest in the three experiments (2.0%, 0.9%, 0.5%).

## Discussion

Good et al [5] applied knowledge-based methods to the IMRT planning of prostate. The results showed improved target homogeneity, sparing of OARs and superior or equivalent plans in 95% of the cases when compared to manual planning. Data demonstrated also the possibility to transfer the planning knowledge from a more to a less experienced institute. Moore et al [6] showed improved plan quality, reduced inter-clinician variability and suggested that the automatic tools might be used as a quality assurance method for IMRT planning.

The scope of the present study was to appraise the possibility to use a knowledge-based dose-constraint prediction methodology with clinically acceptable results. No special efforts were put to analyze in detail the algorithms which are not fully accessible to the investigators. The results obtained for HCC and RapidArc support the conclusion that, even in the case of a relatively ill-defined problem, it was possible to train a dose-volume constraints prediction model with a limited number of patients.

The “ill-defined” condition derived from the variability of tumor sizes, localization in the liver and dose prescriptions resulting in a very large variance of the input data. This was confirmed by the number of outlier candidates identified in the training. All these candidates were classified as influential points and were left in the model after individual examination. Further studies will be needed to determine simple and pragmatic methods to exclude those cases that could significantly (and negatively) impact on the model prediction capability. To notice that the training validation report does not identify the presence of “absolute” outliers but points to the cases where one or more metrics results out of some (arbitrary) threshold and would require further investigation.

The model was built with 45 patients. It was out of scope to determine what could be the minimum reasonable sample size; further studies will address this point. The manufacturer defined a minimum of 20 cases necessary to build a model (in addition, if any given structure would not have at least 20 cases, no prediction model would be provided for it). In addition, the end of training report suggests a recommended number of cases per structure (roughly estimated by the manufacturer to be 5 times the parameters used for the regression model not based on specific metrics). This last value is anyway not mandatory to operate the system and the results suggest that the training can be performed also starting from smaller samples.

Concerning the proper validation, the experiments proved that the model was capable to reproduce the clinical data in both the closed-loop and open-loop validations. On average, model-based plans slightly improved the clinical correspondents. To test the power of generalization of the predictive engine, it was hypothesized that the beam arrangement might be a limiting factor. The results obtained from the open-loop validation II improved the results of the clinical and open-loop I plans. This might be due to external factors including that the non-coplanar setting was not mandatory with the latest version of the optimizer.

The assessment of the quality of the original beam arrangement wasn’t part of the scope of the study but, while it was part of it the investigation of the model robustness to different conditions. As a side effect the data resulted preferable (even if the improvement would likely be not clinically relevant). Within the limits of the study and with the need of further proof, these findings might suggest that the model-based optimization engine might be insensitive to some extent to the geometry chosen, provided that the latter is anyway appropriate. Since, in an inverse planning environment, many different beam arrangement could lead to similar dose distributions through optimization, this result is reasonable.

What observed suggests also the possibility to introduce feed-back loops in the training. The idea would be to replace original training plans with the model-based improved ones and to re-train the model. This might lead to a tightening of the prediction ranges and was proven by the Duke and Washington University groups. We omitted this possibility given the primary goal of the study aiming to demonstrate the basic performance of the system.

A remark concerns the level of “automation” that can be desirable in a model-based system. The predicted dose-volume constraints are located at the lower limit of the predicted range of the possible solutions for the individual case. Priorities for the constraints are automatically generated to guarantee a reasonable probability of achieving the result but all is “constructed” before the actual optimization is performed. It is still conceivable that further improvements could be obtained and/or some complex trade-off should be addressed. Therefore some interactive adjustment of the parameters might be necessary during optimization. In the present study, only the optimization priorities were adjusted for some of the constraints (mostly the maximum target dose or less involved structures) and not all the patients. Data are insufficient to conclude that this procedure is a needed step. In the specific case, it proved to be beneficial but might be that in simpler cases it would be avoided. It was out of the scope of this study to present also an additional comparison with completely un-tweaked priorities, or to fine tune the priorities to set for eventual additional constraints during the model configuration.

The time needed to prepare a model or to generate the constraints for individual patients was briefly addressed and resulted short. The time needed to prepare an optimization and to execute it was not included in the assessment since strongly dependent on the planner skills and, for this reason not easy to objectively quantify. On the other side, the pure optimization time, in the absence of interactive intervention, is independent from the method chosen to generate the dose-volume constraints.

## Conclusions

The construction and training of a knowledge-based dose-volume constraints predictive model was tested successfully for patients with HCC. The quality of the RapidArc plans optimized using this method was consistent compared to the treated plans. Although more systematic studies are needed before a broad clinical application of the methodology, the model-based optimization strategy might be considered as a tool to streamline and improve the planning process.
